# Added value of serial troponin I and N-terminal pro B-type natriuretic peptide for early detection of cardiotoxicity in breast cancer: A three-year retrospective cohort study

**DOI:** 10.5339/qmj.2026.60

**Published:** 2026-09-08

**Authors:** Benard Shehu, Klerida Shehu, Bledar Kraja, Fatjona Kraja

**Affiliations:** 1 Department of Cardiology, University Hospital Center "Mother Teresa," Tirana, Albania; 2 Department of Biomedical and Experimental Sciences, Faculty of Medical Sciences, University of Medicine, Tirana, Albania; 3 Department of Paraclinical Sciences, Faculty of Medical Sciences, University of Medicine, Tirana, Albania; 4 Department of Oncology, University Hospital Center "Mother Teresa," Tirana, Albania

**Keywords:** Cardiovascular toxicity, breast neoplasms, troponin I, natriuretic peptide, echocardiography, Albania

## Abstract

**Background:**

Cardiotoxicity remains a major limitation in breast cancer treatment, especially with anthracyclines and radiotherapy. Early biomarkers may improve risk stratification.

**Methods:**

This retrospective cohort study included breast cancer patients treated between 2020 and 2023 with chemotherapy and/or radiotherapy. Troponin I (TPI) and N-terminal pro B-type natriuretic peptide (NT-proBNP) were measured at baseline and at three, six, 12, 18, 24, 30, and 36 months. Cardiotoxicity was defined per European Society of Cardiology (ESC) guidelines as a ≥10% drop in left ventricular ejection fraction (LVEF) to <53% or >15% relative reduction in global longitudinal strain (GLS). Receiver operating characteristic (ROC) analysis, logistic regression, and mixed-effects models assessed associations.

**Results:**

A total of 190 patients were included; 141 patients (74.2%) developed cardiotoxicity within 12 months. At six months, NT-proBNP (median: 212.6 pg/mL) and TPI (0.038 ng/mL) were significantly higher in the cardiotoxic group (*p* < 0.01). ROC area under the curve (AUC) for NT-proBNP at six months was 0.83 (95% CI: 0.77–0.89); for TPI, AUC = 0.79 (95% CI: 0.73–0.86). Multivariable regression showed both biomarkers remained independent predictors of cardiotoxicity after adjustment for GLS and cumulative anthracycline dose (NT-proBNP: OR 1.78 [95% CI 1.22–2.61], *p* = 0.003).

**Conclusion:**

In this real-world breast cancer cohort, serial NT-proBNP and TPI monitoring, particularly at 3 and 6 months, provided meaningful early prognostic information for subsequent cardiotoxicity beyond baseline assessment alone. When integrated with echocardiographic surveillance, these biomarkers may support earlier identification of high-risk patients, more tailored cardio-oncology follow-up, and timely cardioprotective intervention, although prospective external validation is required before routine implementation.

## 1. INTRODUCTION

Anthracycline-based chemotherapy and human epidermal growth factor receptor 2 (HER2)-targeted agents have significantly improved survival in breast cancer, but their use is limited by potential cardiotoxicity. Cardiac dysfunction may develop insidiously and progress to symptomatic heart failure, even years after therapy. The European Society of Cardiology (ESC) defines cancer therapy-related cardiac dysfunction (CTRCD) as a ≥10% decline in left ventricular ejection fraction (LVEF) to <53%, or a >15% reduction in global longitudinal strain (GLS).Traditional imaging markers may not detect subclinical injury in time to enable preventive strategies.^1^

Cardiac biomarkers such as troponin I (TPI), which reflects cardiomyocyte necrosis, and N-terminal pro B-type natriuretic peptide (NT-proBNP), which indicates myocardial stress, have emerged as adjunctive tools for early detection.^2^ Previous studies have suggested that elevations in these markers during chemotherapy are associated with future left ventricular dysfunction.^3–6^ However, prospective data evaluating the additive prognostic value of serial biomarker trends in real-world populations remain limited. While such biomarker associations are increasingly reported, there is limited real-world evidence on their incremental value in routine clinical practice.^7,8^ This study aimed to assess whether NT-proBNP and TPI predict cardiotoxicity more effectively than baseline values alone, and whether they add independent prognostic information to standard echocardiographic monitoring using GLS and LVEF. We hypothesize that early elevations in these biomarkers during treatment are predictive of both early and persistent myocardial dysfunction.

## 2. MATERIALS AND METHODS

This retrospective cohort study was conducted at the University Hospital Center “Mother Teresa,” Tirana, Albania, between January 2020 and December 2023, and included breast cancer patients treated with chemotherapy and/or radiotherapy.

### 2.1. Inclusion criteria

Inclusion criteria were adult female patients (≥18 years) with histologically confirmed breast cancer treated with anthracyclines and/or radiotherapy and with serial TPI and NT-proBNP measurements available during follow-up.

### 2.2. Exclusion criteria

Exclusion criteria were incomplete biomarker data, uncontrolled arrhythmia, recent myocardial infarction, and pre-existing heart failure (baseline LVEF <50%).

### 2.3. Global longitudinal strain and cardiotoxicity definition

GLS was defined as the echocardiographic measure of myocardial deformation. Cardiotoxicity was defined as an LVEF decline ≥10% to <53% and/or a >15% relative reduction in GLS from baseline, in line with ESC cardio-oncology guidance.

### 2.4. Outcomes

The primary outcome was cardiotoxicity at 12 months, defined by ESC 2022 cardio-oncology guidelines^1^ as: LVEF decline ≥10% to a value <53% or GLS relative reduction >15% from baseline. Secondary outcomes included persistent cardiotoxicity at 36 months and hospitalization for heart failure.

### 2.5. Statistical analysis

Continuous variables were expressed as mean ± SD or median (IQR), and categorical data as counts (%). Group comparisons used Student’s t-test, Mann–Whitney U, or Chi-square tests. Receiver-operating characteristic (ROC) analysis calculated the area under the curve (AUC) for NT-proBNP and TPI at three and six months to predict one-year cardiotoxicity. Logistic regression models were used to determine independent predictors, adjusted for age, GLS, anthracycline dose, and baseline EF. Longitudinal biomarker trends were assessed with mixed-effects linear models. Statistical significance was set at *p* < 0.05. Analyses were performed using SPSS v27 and R v4. three.

## 3. RESULTS

### 3.1. Baseline characteristics 

Among the 190 breast cancer patients included, the mean age was 56.3 ± 8.2 years, with a predominance of cardiovascular comorbidities, including hypertension (45.8%), diabetes mellitus (14.7%), and chronic kidney disease (10%). Anthracycline-based chemotherapy was administered to all patients, and 61.1% also received chest radiotherapy, primarily on the left side (62.1%). Patients who developed cardiotoxicity (*n* = 141, 74.2%) were significantly older (*p* = 0.014) and more frequently had hypertension (*p* = 0.034), diabetes (*p* = 0.045), and received higher cumulative anthracycline doses (*p* = 0.021), as shown in Table 1.

### 3.2. Incidence and definition of cardiotoxicity 

Cardiotoxicity occurred in 141 patients (74.2%) during the first year of follow-up. A GLS decline >15% was the most common criterion (64%), while a reduction in LVEF below 53% accounted for 36% of cases. At 36 months, 25.3% (*n* = 48) had persistent cardiac dysfunction.

### 3.3. Biomarker levels and diagnostic accuracy

**Figure 1.**
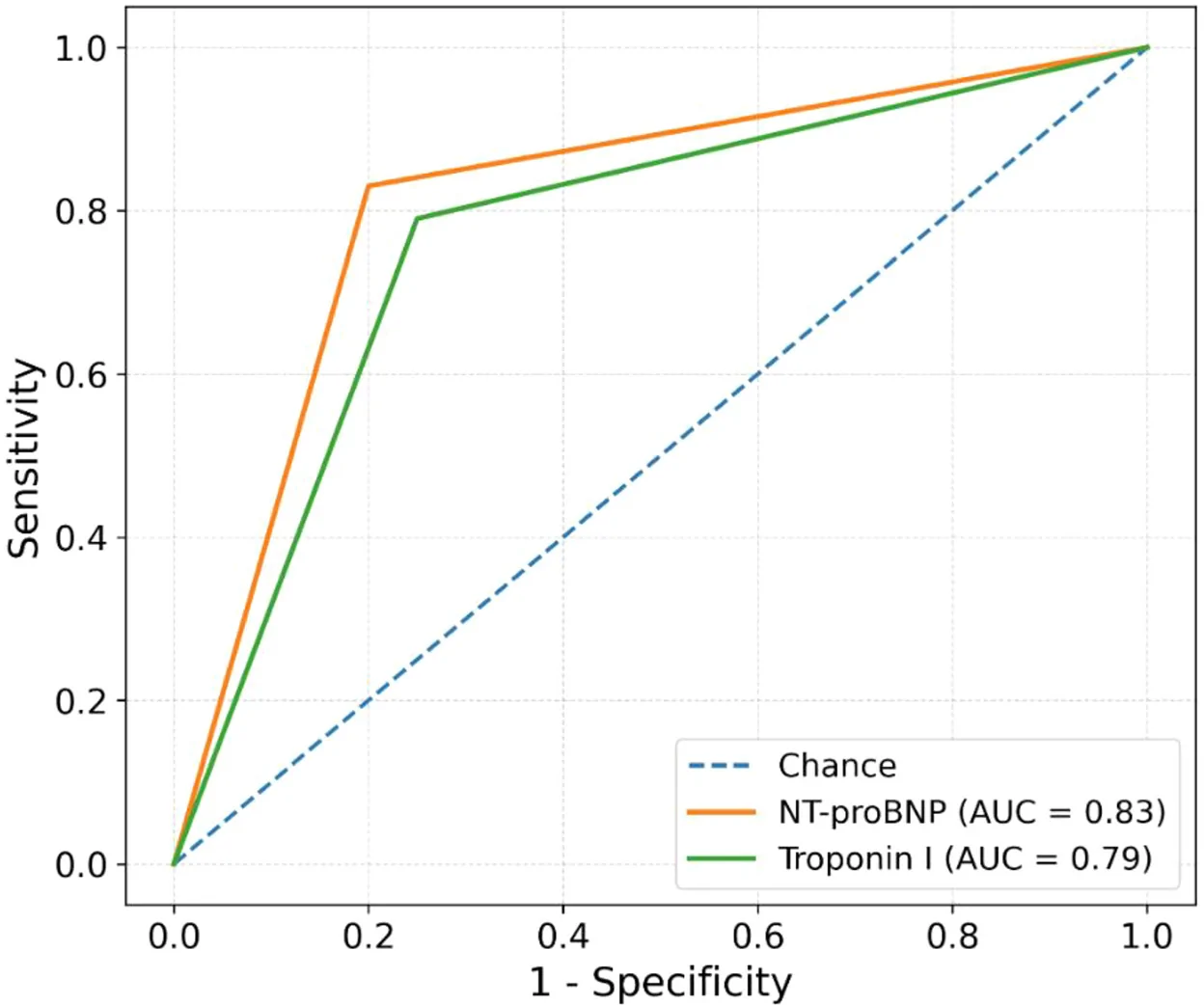
Receiver operating characteristic curves at 6 months for predicting cardiotoxicity in breast cancer patients receiving anthracycline-based therapy.

By the six-month mark, both biomarkers showed significant differences between groups. Patients with cardiotoxicity had higher NT-proBNP (212.6 pg/mL vs. 129.1 pg/mL, *p* < 0.001) and TPI (0.038 ng/mL vs. 0.021 ng/mL, *p* = 0.003). ROC analysis (Table 2) demonstrated high diagnostic performance: AUC for NT-proBNP was 0.83 (95% CI: 0.77–0.89) and for TPI was 0.79 (95% CI: 0.73–0.86) at six months. These findings are illustrated in Figure 1.

### 3.4. Multivariate predictors of cardiotoxicity

In logistic regression models adjusted for clinical and treatment-related variables, both NT-proBNP and TPI retained independent predictive value. As shown in Table 3, a 100 pg/mL increase in NT-proBNP was associated with 1.78-fold higher odds (95% CI: 1.22–2.61, *p* = 0.003) of cardiotoxicity, while each 0.01 ng/mL rise in TPI conferred a 44% increased risk (OR = 1.44, 95% CI: 1.11–1.87, *p* = 0.009). Additionally, anthracycline dose ≥250 mg/m^2^ was a significant covariate (OR = 1.92, *p* = 0.021).

### 3.5. Longitudinal trends in biomarker evolution 

**Figure 2.**
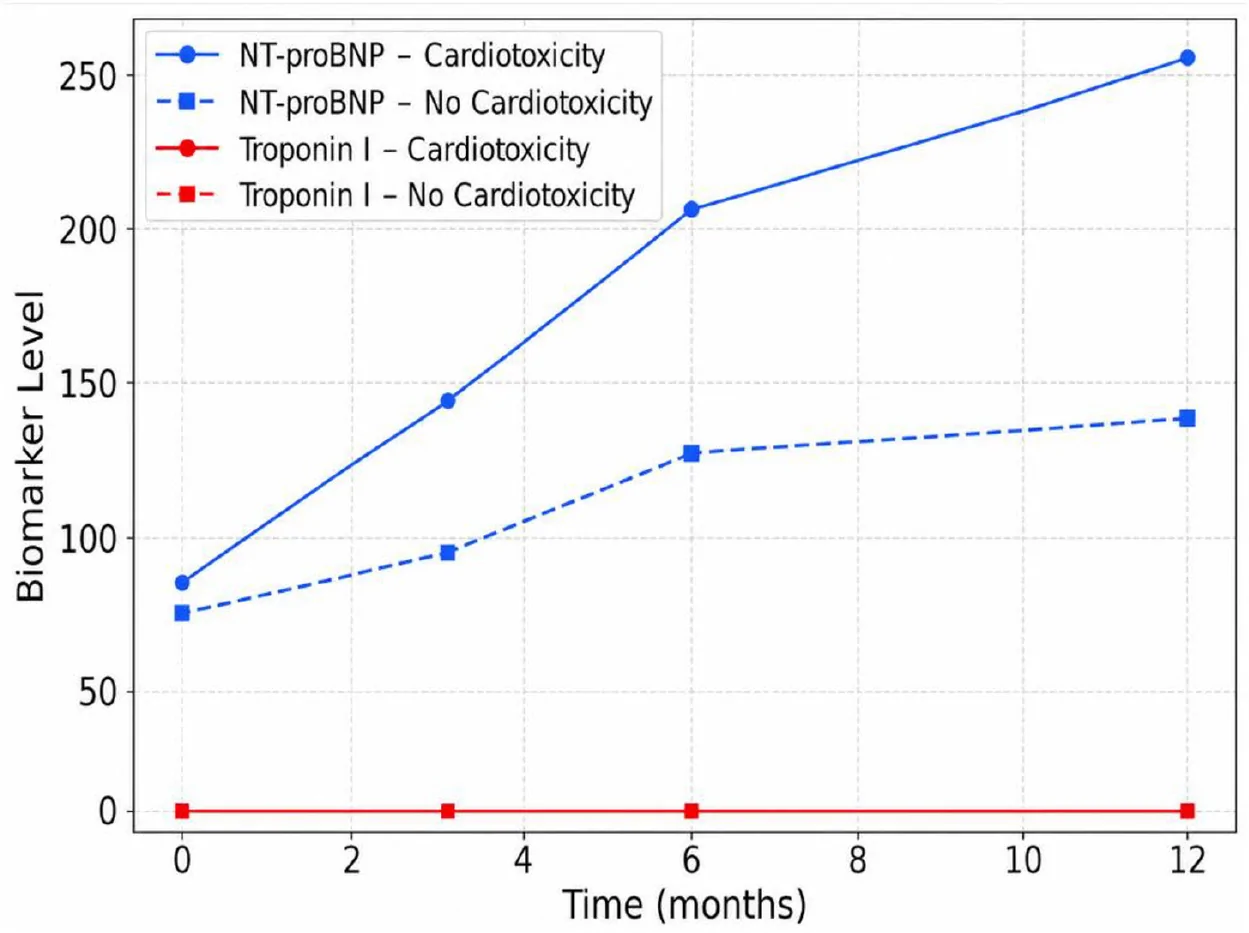
Longitudinal trends of NT-proBNP and TPI over 12 months, by cardiotoxicity status.

**Figure 3.**
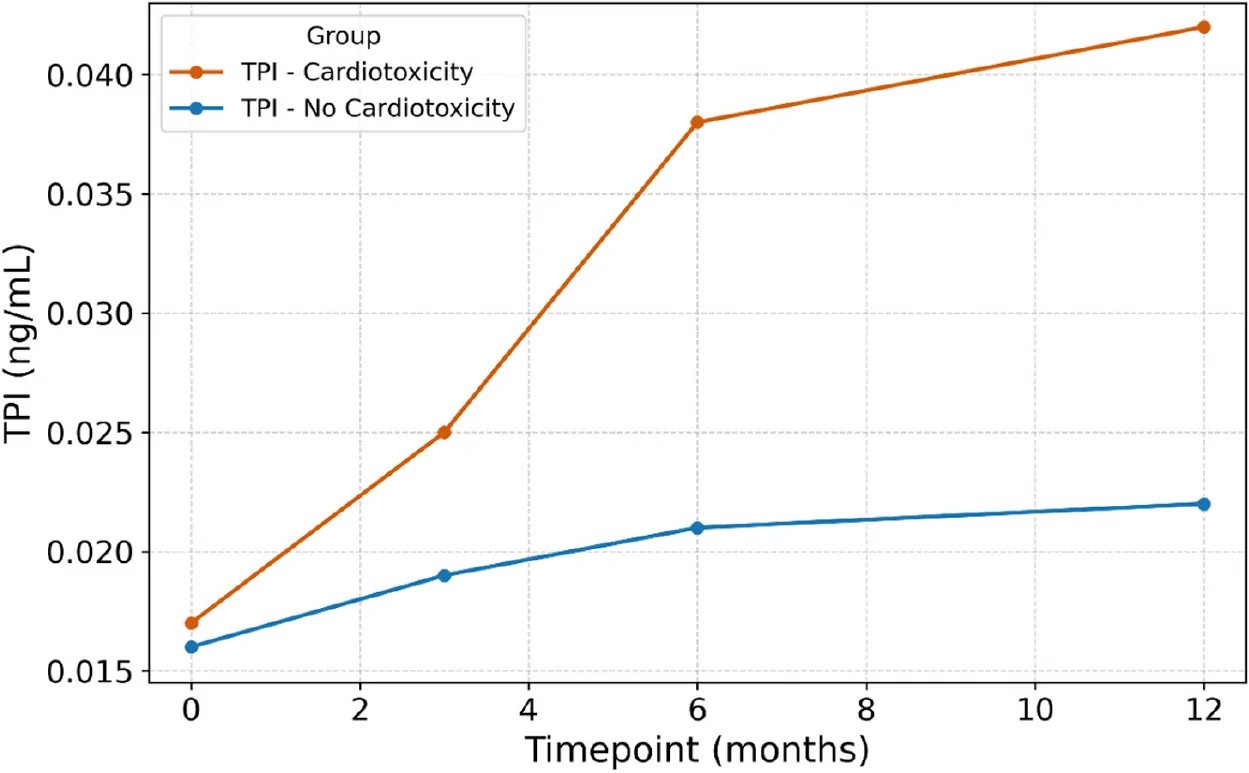
Longitudinal TPI trends over 12 months, by cardiotoxicity status.

**Figure 4.**
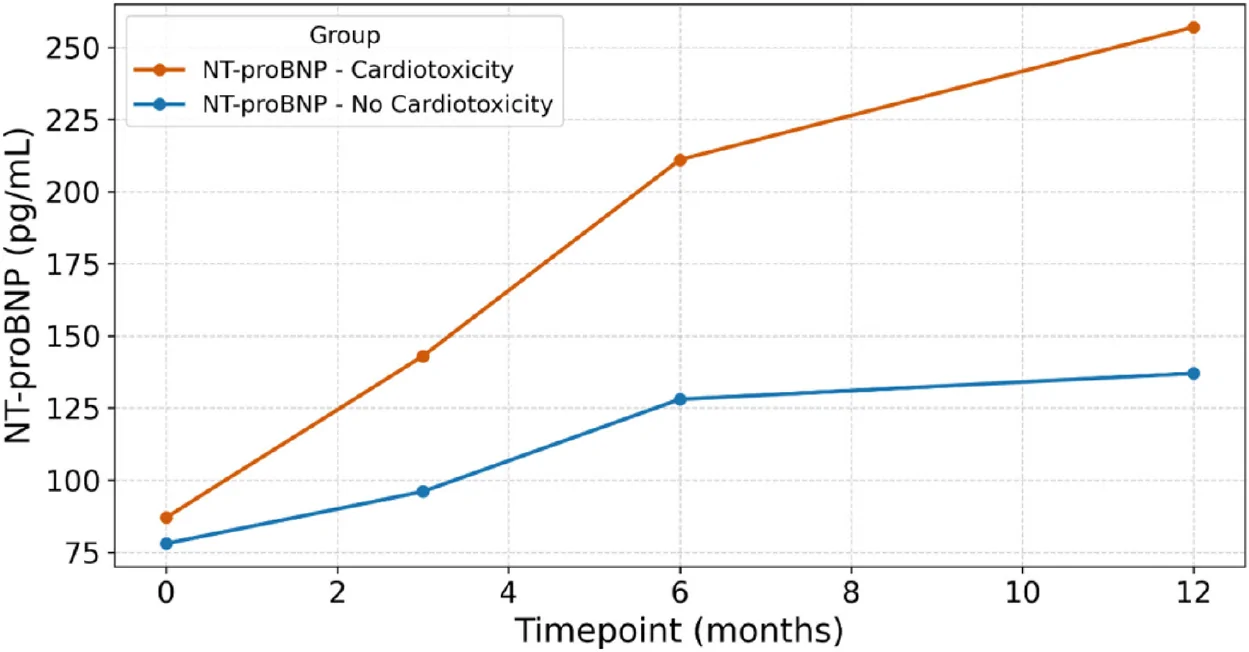
Longitudinal trends of NT-proBNP over 12 months, by cardiotoxicity status.

As shown in Table 4 and visualized in Figures 2–4, NT-proBNP and TPI increased steadily in the cardiotoxicity group over the 12-month period, while levels remained comparatively stable in the non-cardiotoxic group (*p* < 0.001) for time-by-group interaction. NT-proBNP rose from 86.5 pg/mL to 256.2 pg/mL in the affected group, while TPI increased from 0.017 to 0.042 ng/mL, showing progressive biomarker elevation over time.

### 3.6. Correlation with echocardiographic markers

**Figure 5.**
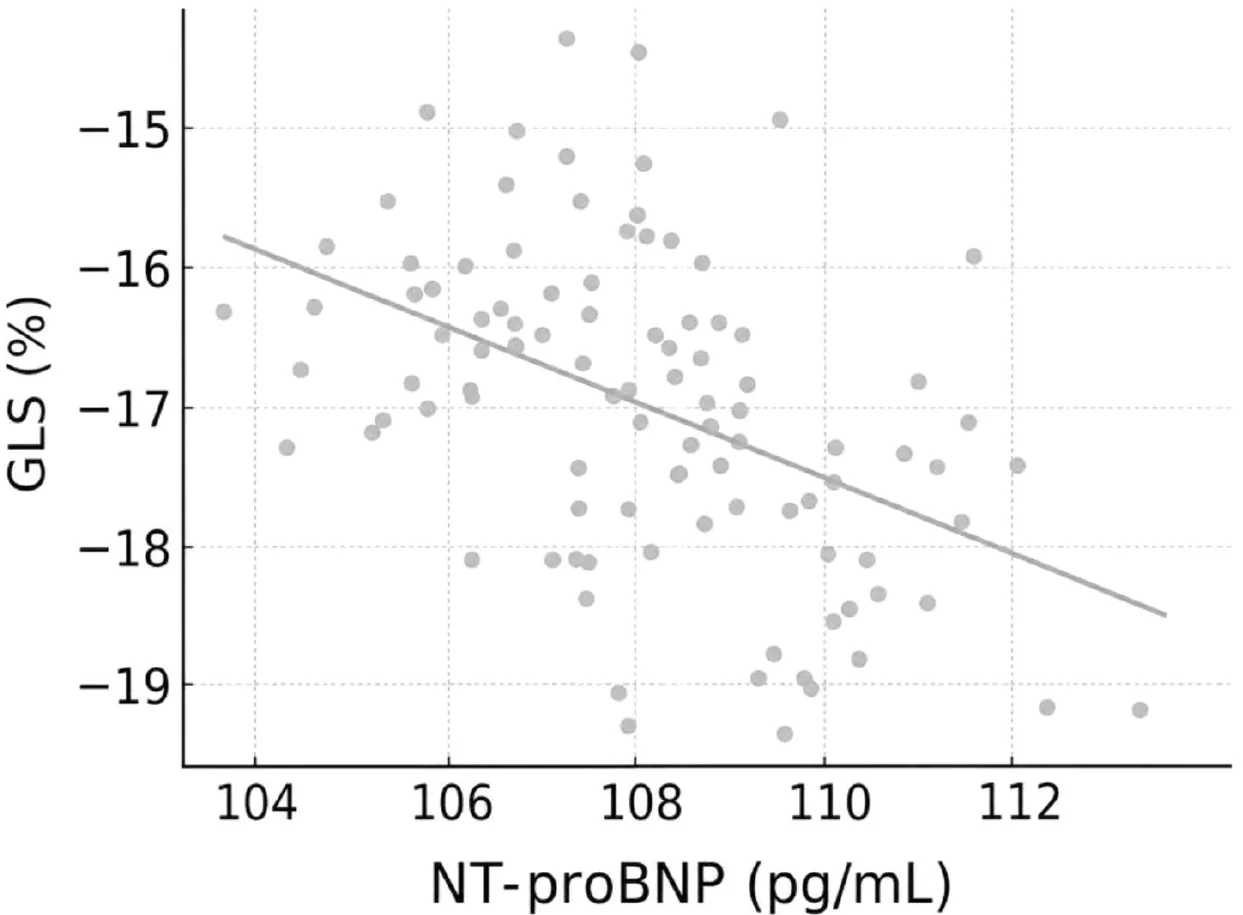
Correlation between NT-proBNP and GLS at 6 months.

At six months, NT-proBNP levels inversely correlated with GLS (*r* = –0.48, *p* < 0.001), suggesting that elevated biomarker values reflect impaired myocardial deformation. This association is detailed in Table 5 and illustrated in Figure 5.

### 3.7. Alternative cut-off approaches 

**Figure 6.**
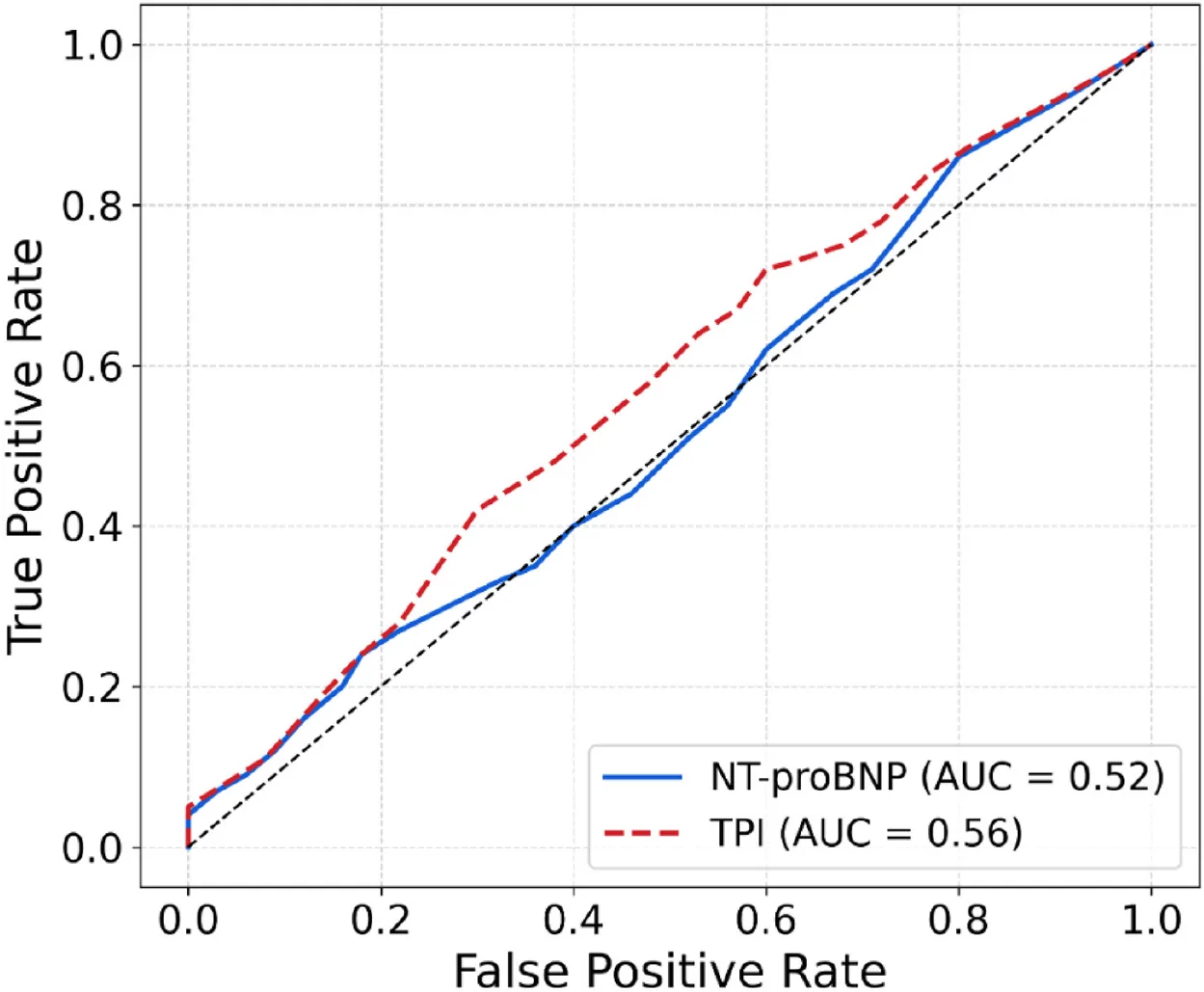
Receiver operating characteristic curves at 6 months using alternative cut-off approaches.

Exploratory ROC analyses using optimized cut-offs, including Youden’s index and relative changes from baseline, showed similar diagnostic accuracy to the primary analysis. These analyses showed similar diagnostic performance to the primary analysis, as shown in Figure 6.

## 4. DISCUSSION

Our results align with those reported by Cardinale et al.^4^ who first demonstrated that troponin elevation predicts left ventricular dysfunction in breast cancer patients undergoing anthracyclines and trastuzumab therapy. Similarly, Ky et al.^5^ confirmed that early biomarker rise correlates with later cardiotoxicity. In contrast to those prospective cohort studies, our retrospective real-world data reaffirm the prognostic strength of troponin I and NT-proBNP. Strain imaging via GLS, as advocated by Thavendiranathan et al.^6^ remains a cornerstone of early dysfunction assessment; however, our findings show that NT-proBNP independently correlates with GLS, echoing the mechanistic insights of Michel et al.^7^ The superiority of NT-proBNP over TPI in our ROC analysis supports observations by Putt et al.^8^ who showed longitudinal NT-proBNP elevations more closely track with structural dysfunction. Finally, the complementary utility of echocardiography and biomarkers is consistent with the integrative surveillance models proposed by Sawaya et al.^9^

The present study provides robust evidence supporting the clinical value of serial TPI and NT-proBNP measurements for the early identification of cardiotoxicity in breast cancer patients undergoing anthracycline-based chemotherapy and/or chest radiotherapy. Our results corroborate prior findings from smaller prospective cohorts but expand the evidence by demonstrating that serial rather than isolated biomarker measurements enhance predictive accuracy, particularly when integrated with imaging modalities like GLS.^10^

The high incidence of cardiotoxicity observed in our cohort (74.2%;Table 1) reflects both the aggressive oncologic regimens used and the rigorous definition of subclinical myocardial dysfunction according to ESC 2022 guidelines.^1^ Importantly, over one quarter of patients exhibited persistent cardiac dysfunction at 36 months, underscoring the clinical relevance of long-term cardiac surveillance beyond the acute treatment window.

NT-proBNP emerged as the most reliable early predictor of cardiotoxicity, with an AUC of 0.83 at six months (Table 2, Figure 1). This finding aligns with its established role in detecting myocardial wall stress and subclinical diastolic dysfunction, often preceding systolic decline. Our study strengthens its application in cardio-oncology by demonstrating its longitudinal association with GLS reduction, a sensitive echocardiographic marker of myocardial deformation.^11^

Similarly, the role of TPI in reflecting cardiomyocyte necrosis was confirmed through its moderate discriminative ability (AUC = 0.79; Table 2, Figure 1), consistent with prior trials such as the OVERCOME study (prevention of left ventricular dysfunction with enalapril and carvedilol in patients submitted to intensive chemotherapy for the treatment of malignant hemopathies).^12^ Interestingly, TPI and NT-proBNP showed complementary value, supporting the hypothesis that combining necrotic and hemodynamic markers yields superior risk stratification.

Our multivariate models confirm that biomarker dynamics remain independently associated with cardiotoxicity even after adjusting for known confounders including baseline GLS and cumulative anthracycline dose (Table 3).^13^ Furthermore, the significance of the mixed-effects model in revealing time–treatment interactions illustrates the dynamic evolution of cardiac injury, which might be overlooked by conventional cross-sectional assessments.

The progressive biomarker elevation over 12 months observed in the cardiotoxicity group (Table 4; Figures 2–4) supports the concept of ongoing myocardial stress/injury during and after treatment and provides a mechanistic rationale for closer follow-up in higher-risk patients.

### 4.1. Clinical implications

Our findings suggest that routine serial monitoring of NT-proBNP and TPI can enhance the early detection of cardiotoxicity in breast cancer patients receiving anthracycline-based therapy. Incorporating these biomarkers alongside GLS assessment may facilitate timely risk stratification and targeted cardio-oncology interventions in line with ESC recommendations.

In our cohort, diagnostic performance at 6 months is summarised in Table 2 and Figures 1 and ^6^, while longitudinal biomarker trajectories are reported in Table 4 and Figures 2–4; the association between NT-proBNP and GLS is shown in Figure 5.

In clinical terms, our findings advocate for the integration of serial biomarker monitoring into existing imaging-based protocols.^14^ This is particularly relevant in settings where GLS assessment is technically limited or resource-constrained. Incorporating threshold-based decision-making for NT-proBNP and TPI may enable earlier cardioprotective interventions such as the administration of ACE inhibitors or beta-blockers.^15,16^

### 4.2. Limitations

Considering our study, however, several limitations must be acknowledged. First, the retrospective design may introduce selection bias, although uniform treatment protocols and centralized biomarker testing mitigate this risk. Second, the study population was limited to a single institution, potentially affecting generalizability. Third, biomarker thresholds were not pre-specified but derived empirically, warranting validation in external cohorts.

Future research should aim at prospective external validation in multi-center cohorts, including predefined sampling timepoints and assay standardization. Studies should also test whether biomarker-guided surveillance and early cardioprotective management improve clinically relevant outcomes (e.g., fewer treatment interruptions, fewer heart failure admissions, and better preservation of left ventricular function). Finally, comparative work combining biomarkers with echocardiographic strain and clinical risk scores could support practical, resource-sensitive prediction models.

## 5. CONCLUSION

In this Albanian breast cancer cohort, serial TPI and NT-proBNP—particularly at 3 and 6 months—were independently associated with subsequent cardiotoxicity. These findings support a pragmatic biomarker-based monitoring strategy to identify higher-risk patients earlier and to trigger intensified echocardiographic surveillance and timely cardio-protective management when indicated, to preserve cardiac function and avoid treatment interruptions. Prospective external validation is needed before routine implementation.

## ACKNOWLEDGEMENT

The authors thank the clinical staff involved in patient care and data collection.

## AUTHORS’ CONTRIBUTIONS

BS: Conceptualization, methodology, data curation, software, formal analysis, investigation, visualization, and drafting of the original manuscript. KS: Methodology, supervision, validation, and critical revision of the manuscript for important intellectual content. BK: Data curation, formal analysis, validation, and interpretation of the results. FK: Clinical data acquisition, investigation, and interpretation of oncological variables. All authors contributed to manuscript review and editing, approved the final version of the manuscript, and agreed to be accountable for all aspects of the work.

## CONFLICT OF INTEREST

The authors declare no conflicts of interest.

## DISCLOSURE OF AI USE

Generative AI and language-assistance tools, including ChatGPT, Claude, and Grammarly, were used as supportive tools during manuscript preparation, primarily for English-language drafting, editing, and stylistic refinement. These tools were not used for data collection, statistical analysis, interpretation of results, or scientific decision-making. The authors reviewed and approved the final content and take full responsibility for the manuscript.

## DATA AVAILABILITY

The data that support the findings of this study are available from the corresponding author upon reasonable request.

## FUNDING

The author(s) received no financial support for the research, authorship, and/or publication of this article.

## ETHICAL APPROVAL

The study was conducted in accordance with the Declaration of Helsinki and approved by the institutional ethics committee (Decision No. 11 - 18 03 2024). Due to the retrospective design and use of de-identified clinical data, the requirement for individual informed consent was waived.

## CONSENT FOR PUBLICATION

Not applicable.

## Figures and Tables

**Table 1.** Baseline demographic and clinical characteristics of the study population stratified by cardiotoxicity status.

Variable	Total (*n* = 190)	Cardiotoxicity (*n* = 141)	No Cardiotoxicity (*n* = 49)	*p*-value
Age (years), median [IQR]	55 [48–63]	57 [50–65]	51 [44–60]	0.014
Hypertension, *n* (%)	87 (45.8%)	71 (50.4%)	16 (32.7%)	0.034
Diabetes Mellitus, *n* (%)	28 (14.7%)	24 (17.0%)	4 (8.2%)	0.045
Chronic Kidney Disease, *n* (%)	19 (10.0%)	17 (12.1%)	2 (4.1%)	0.041
Anthracycline Dose ≥250 mg/m^2^, *n* (%)	104 (54.7%)	87 (61.7%)	17 (34.7%)	0.021
Left-sided Radiotherapy, *n* (%)	72 (62.1%)	58 (66.7%)	14 (50.0%)	0.037

**Table 2.** Receiver operating characteristic analysis of biomarkers at 6 months for predicting cardiotoxicity in breast cancer patients receiving anthracycline-based chemotherapy and/or chest radiotherapy.

Biomarker	Timepoint	AUC (95% CI)	*p*-value
NT-proBNP	6 months	0.83 (0.77–0.89)	<0.001
TPI	6 months	0.79 (0.73–0.86)	0.003

**Table 3.** Multivariable logistic regression predictors of 12-month cardiotoxicity in breast cancer patients treated with anthracyclines and/or chest radiotherapy.

Variable	Odds Ratio (OR)	95% CI	*p*-value
NT-proBNP (per 100 pg/mL)	1.78	1.22–2.61	0.003
Troponin I (per 0.01 ng/mL)	1.44	1.11–1.87	0.009
Anthracycline Dose ≥250 mg/m^2^	1.92	1.12–3.41	0.021
GLS at baseline (% per unit)	0.88	0.81–0.96	0.005
Hypertension	1.62	1.01–2.59	0.046

**Table 4.** Longitudinal trends of NT-proBNP and TPI over 36 months in breast cancer patients treated with anthracycline-based chemotherapy and/or chest radiotherapy.

Timepoint (months)	NT-proBNP – Cardiotoxicity	NT-proBNP – No Cardiotoxicity	TPI – Cardiotoxicity	TPI – No Cardiotoxicity
Baseline	86.5 ± 29.3	78.1 ± 25.6	0.017 ± 0.005	0.016 ± 0.004
3	143.4 ± 34.7	96.2 ± 31.1	0.025 ± 0.007	0.019 ± 0.005
6	212.6 ± 39.8	129.1 ± 33.4	0.038 ± 0.010	0.021 ± 0.006
12	256.2 ± 41.1	138.5 ± 37.2	0.042 ± 0.011	0.022 ± 0.007

**Table 5.** Correlation of GLS with biomarker levels at six months.

Biomarker	Pearson (r) with GLS	*p*-value
NT-proBNP at 6 months	–0.48	<0.001
TPI at 6 months	–0.37	0.004
